# Early Normalization of Alanine Aminotransferase during Antiviral Therapy Reduces Risk of Hepatocellular Carcinoma in HBV Patients

**DOI:** 10.3390/jcm10091840

**Published:** 2021-04-23

**Authors:** Sehwa Kim, Yoonseok Lee, Soo Min Bang, Haein Bak, Sun Young Yim, Young Sun Lee, Yang Jae Yoo, Young Kul Jung, Ji Hoon Kim, Yeon Seok Seo, Hyung Joon Yim, Soon Ho Um, Kwan Soo Byun, Jong Eun Yeon

**Affiliations:** 1 Department of Internal Medicine, Korea University College of Medicine, Seoul 08308, Korea; ms2844579@gmail.com (S.K.); seok7288@gmail.com (Y.L.); soomin1987@naver.com (S.M.B.); alien2719@naver.com (H.B.); eug203@naver.com (S.Y.Y.); lys810@korea.ac.kr (Y.S.L.); 93cool@hanmail.net (Y.K.J.); kjhhepar@naver.com (J.H.K.); gandorie@gmail.com (Y.S.S.); gudwns21@korea.ac.kr (H.J.Y.); umsh@korea.ac.kr (S.H.U.); kwsbyun@unitel.co.kr (K.S.B.); 2 Department of Internal Medicine, Bundang Jesaeng General Hospital, Seongnam-si 13590, Korea; tianmu0627@gmail.com

**Keywords:** alt normalization, hcc, entecavir, tenofovir

## Abstract

Potent antiviral agents effectively reduce liver-related events in patients with chronic hepatitis B. This study aimed to determine whether alanine aminotransferase normalization using potent antiviral agents was related to hepatocellular carcinoma development. From 2007 to 2017, we included 610 patients with chronic hepatitis B who received entecavir or tenofovir disoproxil fumarate. The patients were divided into the alanine aminotransferase normalization group (Gr.1) and non-normalization group (Gr.2) within a year of potent antiviral treatment. Liver-related events included hepatic encephalopathy, variceal bleeding, and ascites. The mortality rate and hepatocellular carcinoma incidence were investigated for each group. The patients who showed ALT normalization at 1 year of treatment were 397 (65.1%) of 610. During a median follow-up period of 86 months, 65 (10.7%) patients developed hepatocellular carcinoma. The cumulative incidence of hepatocellular carcinoma was significantly lower in Gr.1 than in Gr.2 (*p* < 0.001). Risk factors for alanine aminotransferase non-normalization were body mass index, cholesterol, and liver cirrhosis at baseline. Male sex, age, platelet level, alcohol use, presence of cirrhosis at baseline, and non-normalization after 1 year of treatment were independent risk factors for hepatocellular carcinoma. Alanine aminotransferase normalization within 1 year of initiating antiviral agents reduces the risk of hepatocellular carcinoma development.

## 1. Introduction

An estimated 240 million people worldwide are chronically infected with hepatitis B virus (HBV) [1]. HBV infection is the second most common cause of mortality worldwide and the most common cause of liver cancer [2,3]. Although the prevalence of chronic hepatitis B (CHB) is decreasing since the introduction of neonatal vaccination, many developing countries still have a high risk of hepatocellular carcinoma (HCC) development [4].

In the clinical course of CHB, decreased HBV DNA levels, serum alanine aminotransferase (ALT) normalization, and hepatitis B envelope antigen (HBeAg) seroconversion have been associated with HCC and mortality [5,6]. Patients with high serum HBV DNA levels or high levels of ALT have a higher risk of developing HCC [5,7]. In patients with HBV, additional risk factors, including sex, age, smoking, alcohol, chemical agents, hormonal factors, and genetic susceptibility contribute to HBV-related HCC development [2].

Recently, the use of potent antiviral agents has made it possible to effectively reduce liver-related events such as hepatic encephalopathy, variceal bleeding, ascites, and HCC in patients with CHB [8]. Effective antiviral agents such as entecavir (ETV) and tenofovir disoproxil fumarate (TDF) have shown to suppress HBV DNA and are recommended as the first-line therapy for CHB [9,10]. In patients with CHB treated with antivirals, virologic responses in which HBV DNA was not detected by PCR or biochemical responses showing ALT normalization demonstrated a lower risk of developing HCC [11]. Thus, the main goal of CHB management is not only to prevent disease progression but also to improve survival through treatment of CHB [9]. Regarding HCC risk in patients with CHB who are being treated with potent antivirals, a few studies showed that early ALT normalization with antiviral agent treatment has a lower risk of developing liver-related events in patients with chronic HBV [12].

This study aimed to determine whether ALT normalization using antiviral agents is related to the occurrence of liver-related events and HCC. We also determined the factors related to ALT normalization and HCC occurrence. 

## 2. Material and Methods

### 2.1. Patients

We collected data from patients with CHB who started treatment with ETV or TDF at Korea University Guro Medical Center in Seoul, Korea, from January 2007 to December 2017. We included patients who were followed up at the hospital for at least 1 year after starting treatment, and they were either antiviral naïve or experienced. The patients were divided into an ALT normalization group and ALT non-normalization group after 1 year of antiviral treatment. We excluded patients who met any of the following criteria: (i) age < 18 years or >80 years, (ii) ALT level within normal standard at baseline, (iii) undetectable serum HBV DNA at baseline, (iv) diagnosed with HCC within 1 year of starting treatment, (v) incomplete medical records (no baseline ALT or HBV DNA data), (vi) follow-up period of less than 1 year, and (vii) coinfection with hepatitis C or hepatitis D virus. Liver-related events included hepatic encephalopathy, variceal bleeding, and ascites.

We obtained approval from the Institutional Review Board (IRB No. 2020GR0258) for this study. The study was conducted in accordance with the Declaration of Helsinki. Informed consent was waived because of the retrospective nature of the study and the analysis used anonymous clinical data.

### 2.2. Clinical and Laboratory Variables

Liver chemistries, including serum ALT, were checked at baseline and at 3–6-month intervals during antiviral treatment. Normal ALT levels were defined as <35 IU/L for men and <25 IU/L for women following the American Association of Study of Liver Disease (AASLD) standard [13]. HBeAg and anti-hepatitis B envelope antibody (HBeAb) were examined by enzyme immunoassay. Serum HBV DNA levels were investigated by real-time polymerase chain reaction (The Roche COBAS^®^ AmpliPrep/COBAS^®^ TaqMan^®^ HBV v2.0 assay, limit of detection <20 IU/mL). Liver cirrhosis was diagnosed when one of the four criteria was met: liver surface nodule findings on ultrasonography, or thrombocytopenia (<100,000/mm^3^), prolongation of prothrombin time (INR > 1.3), or signs of portal hypertension (splenomegaly >120 mm, patent collateral veins, or ascites) [14,15].

HCC was screened based on the ICD-9-CM diagnostic code of radiological or pathological findings, and the diagnosis was confirmed through liver dynamic CT, liver MRI, or liver biopsy.

### 2.3. Events

The primary endpoints were HCC incidence and mortality. Liver-related events, such as ascites, variceal bleeding, and hepatic encephalopathy, were also investigated. The death of a patient was confirmed through the Korean insurance database.

### 2.4. Statistical Analysis

Values are presented as means or medians or as numbers of patients (%). The cumulative incidence of HCC was calculated using the Kaplan–Meier method. To identify the risk factors for ALT non-normalization and HCC, univariate and multivariate analyses were performed by logistic analysis and Cox regression analysis, including clinical variables at baseline. In univariate analysis, if the variable had *p* ≤ 0.1, that variable was used for multivariate analysis. Statistical significance was set at *p* ≤ 0.05. Statistical analyses were conducted using IBM^®^ SPSS^®^ Statistics Version 25.0. Propensity score matching was done using the PSMATCH procedure in SAS version 9.4 with the greedy nearest neighbor match without replacement within 0.2 caliper width. All reported *p* values are two-sided, and *p* < 0.05 was considered statistically significant.

## 3. Results

### 3.1. Patient Characteristics

We analyzed the baseline characteristics of 610 patients. The median age of the patients was 47 years (interquartile range [IQR], 39–55), and 62.6% of the patients were males (Table 1). HBeAg-positive patients constituted 62.1%, and patients naïve to CHB treatment constituted 85.4%. The median HBV DNA level was 5.64 × 10^6^ IU/mL, and the median ALT level was 113 IU/L (IQR, 66–227). Liver cirrhosis was present in 30.5% of the patients, and the median follow-up duration was 86 months (IQR, 35–94).

Among 610 patients, 397 (65.1%) achieved ALT normalization after 1 year of antiviral treatment. Patients with ALT normalization (Gr.1) were younger and had higher baseline platelet counts, and their baseline ALT, aspartate aminotransferase (AST), and HBV DNA titers were also higher than those of non-ALT normalization group (Gr.2). Gr.1 had more patients without cirrhosis and a lower body mass index (BMI) than Gr.2 (Table 1).

Because the coexistence of liver cirrhosis and distribution of BMI were different between the two groups, we performed propensity matching. The baseline characteristics of the 378 study patients after propensity score matching are summarized in Table 2. There were no significant differences in age, platelet counts, ALT levels, AST levels, HBV DNA titer, presence of cirrhosis, and BMI. 

After propensity matching, the follow-up duration was 90 months and 83 months in ALT normalization group (Gr.1) and ALT non-normalization group (Gr.2), respectively. In addition, the ETV use or TDF use rates differed significantly between the two groups. The rate of ETV use was significantly higher in Gr.1 than Gr.2 and the rate of TDF use was significantly higher in Gr. 2 than Gr.1.

### 3.2. LC Development According to ALT Normalization 

In patients without liver cirrhosis at baseline, we aimed to determine whether ALT normalization was related to the occurrence of cirrhosis in the future. A total of 424 patients did not have cirrhosis before treatment, of which 26 developed cirrhosis during the follow-up period. A total of 308 patients (72.6%) showed ALT normalization (Gr.1), and 116 (27.4%) showed ALT non-normalization (Gr.2) after 1 year of treatment. Cirrhosis occurred in 17 (5.5%) of 308 patients with ALT normalization and 9 (7.8%) of 116 patients with ALT non-normalization. 

In Gr.1, the cumulative incidence of cirrhosis were 10 (3.2%), 15 (4.9%), and 16 (5.2%) at 2, 5, and 8 years, respectively. In Gr.2, the cumulative incidence of cirrhosis were 3 (2.6%), 8 (6.9%), and 9 (7.8%) at 2, 5, and 8 years, respectively. After 40 months of starting the antiviral agent, the cumulative incidence of cirrhosis in Gr.2 was numerically higher than that in Gr.1, but it was not significant (*p* = 0.431). 

### 3.3. Liver-Related Event

Liver-related events include hepatic encephalopathy, variceal bleeding, and ascites. During the follow-up period, more than one liver-related event occurred in 36 of the 610 patients in total and 23 of the 378 patients after propensity matching.

In Gr.1, among the 610 patients, the cumulative incidence of liver-related events were 11 (2.8%), 17 (4.3%), and 21 (5.3%) at 2, 5, and 8 years, respectively. In Gr.2, the cumulative incidence of liver-related events were 7 (3.3%), 10 (4.7%), and 13 (6.1%) at 2, 5, and 8 years, respectively (*p* = 0.694). 

After propensity score matching, the cumulative incidence of liver-related events were 6 (3.2%), 10 (5.3%), and 14 (7.4%) at 2, 5, and 8 years, respectively, in Gr.1 and 4 (2.1%), 7 (3.7%), and 9 (4.8%), respectively, in Gr.2 (*p* = 0.404). 

In the analysis of 610 patients, the cumulative incidence of liver-related events of Gr.2 was higher than Gr.1, but the results did not show significant difference. Conversely, after propensity score matching, the cumulative incidence of liver-related events of Gr.1 were higher, but it was also not a significant difference.

### 3.4. Survival 

**Figure 1:**
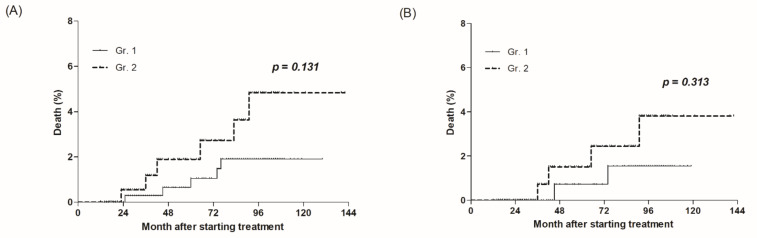
All-cause mortality according to early on-treatment ALT normalization. (**A**) A total of 610 patients, and (**B**) 378 patients after propensity matching.

A total of 11 deaths occurred during the median follow-up period of 86 months (IQR, 35–94). There were 5 of 397 (1.3%) and 6 of 213 (2.8%) deaths in Gr.1 and Gr.2, respectively (*p* = 0.131) (Figure 1A). 

After propensity score matching, the median follow-up period of the 378 patients was 87 months (IQR, 36–96). There were 2 deaths in 189 patients (1.1%) and 4 in 189 (2.1%) in Gr.1 and Gr.2, respectively (*p* = 0.313) (Figure 1B). 

The morality of Gr.2 was higher both before and after propensity score matching, but this was not a significant result.

### 3.5. HCC Development

#### 3.5.1. HCC Incidence in All Patients 

During the follow-up period, 65 (10.7%) of 610 patients developed HCC.

**Figure 2:**
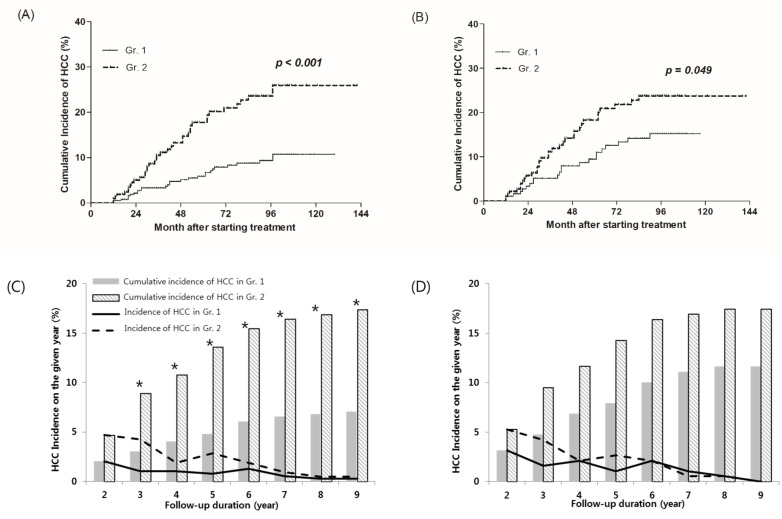
Cumulative incidence of HCC according to early treatment ALT normalization. (**A**,**C**) A total of 610 patients, and (**B**,**D**) 378 patients after propensity matching. HCC, hepatocellular carcinoma. * Denote the columns at the time when significant differences were observed.

**Figure 3:**
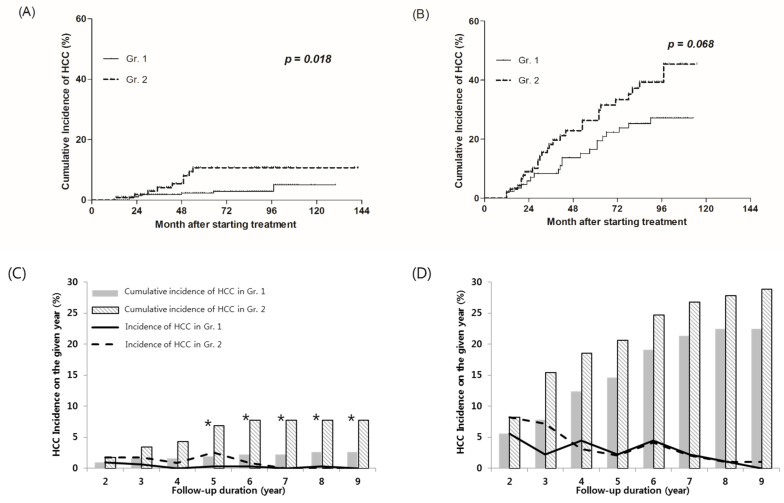
HCC cumulative incidence according to early on-treatment ALT normalization. (**A**,**C**) Patients without liver cirrhosis at baseline, and (**B**,**D**) patients with liver cirrhosis at baseline. HCC, hepatocellular carcinoma. * Denote the columns at the time when significant differences were observed.

The cumulative incidence of HCC at the 2 years after treatment were 2.0% and 4.7% in Gr.1 and Gr.2, respectively. Although it was not a significant difference until this time point (*p* = 0.062), the difference between the two groups has been significant since 2 years. The cumulative HCC incidence of Gr.1 and Gr.2 were 4.8% and 13.6% at 5 years (*p* = 0.001), 6.8% and 16.9% at 8 years, respectively (*p* < 0.001) (Figure 2C). In the Figure 2C,D and Figure 3C,D, the solid and dotted lines show the HCC incidence at each of time points, and the columns show the cumulative incidences. The columns at the time when significant differences were observed are marked with stars (*). The cumulative incidence of HCC estimated by the Kaplan–Meier method was significantly lower in Gr.1 than Gr.2. (*p* < 0.001) (Figure 2A). 

After propensity score matching, HCC occurred in 55 patients (14.6%) out of 378 patients. The cumulative incidence of HCC at the 2 years after treatment were 3.2% and 5.3% in Gr.1 and Gr.2, respectively (*p* = 0.307). The cumulative HCC incidence of Gr.1 and Gr.2 were 7.9% and 14.3% at 5 years (*p* = 0.050), and 11.6% and 17.5% at 8 years, respectively (*p* = 0.109). Although the cumulative incidence of HCC in Gr.1 was lower than Gr.2, no significant difference between the two groups was found at any time point (Figure 2D). The cumulative incidence of HCC was estimated using the Kaplan–Myer method and showed significantly lower incidence of Gr.1 than Gr.2. (*p* = 0.049) (Figure 2B).

#### 3.5.2. HCC Development According to the Presence or Absence of ALT Normalization in Patients without Baseline Liver Cirrhosis

There were 424 patients who did not have liver cirrhosis at baseline. Eight (2.6%) out of 308 patients in the ALT normalization group (Gr.1) and nine (7.8%) out of 116 patients in the ALT non-normalization group (Gr.2) developed HCC. The cumulative incidence of HCC at the 2 years were 1.0% and 1.7% in Gr.1 and Gr.2, respectively (*p* = 0.524); it was not a significant difference until 5 years. The cumulative HCC incidence of Gr.1 and Gr.2 were 1.9% and 6.9% at 5 years (*p* = 0.004), and 2.6% and 7.8% at 8 years, respectively (*p* = 0.008) (Figure 3C). Figure 3A shows the cumulative incidence of HCC using the Kaplan–Myer method. In patients with non-cirrhotic CHB, the cumulative incidence of HCC was significantly lower in Gr.1 than in Gr.2 (*p* = 0.018). 

#### 3.5.3. HCC Development According to the Presence or Absence of ALT Normalization in Patients with Baseline Liver Cirrhosis 

Out of 610 patients, 186 had liver cirrhosis at baseline. Twenty (22.5%) out of 89 patients in the ALT normalization group (Gr.1) and 28 (28.9%) out of 97 patients in the ALT non-normalization group (Gr.2) developed HCC. 

The cumulative incidence of HCC at the 2 years after treatment were 5.6% and 8.2% in Gr.1 and Gr.2, respectively (*p* = 0.482). The cumulative HCC incidence of Gr.1 and Gr.2 were 14.6% and 20.6% at 5 years (*p* = 0.284), and 22.5% and 27.8% at 8 years, respectively (*p* = 0.400). Although the cumulative incidence of HCC in Gr.1 was lower than Gr.2, no significant difference between the two groups was shown at any time point. (Figure 3D). The cumulative incidence of HCC estimated by the Kaplan–Meier method showed lower in Gr.1 than Gr.2, but it was not a significant difference (*p* = 0.068) (Figure 3B).

#### 3.5.4. HCC Development According to HBeAg Seroconversion

In patients who were initially HBeAg-positive, the incidence of HCC, according to HBeAg seroconversion after 1 year of treatment was analyzed. A total of 379 patients were initially HBeAg-positive. Fifty-six (14.8%) of the 379 patients underwent seroconversion 1 year after treatment. 

The cumulative incidence of HCC in the HBeAg-seroconverted group were 2 (3.6%), 5 (8.9%), and 6 (10.7%) at 2, 5, and 8 years, respectively. In the non-seroconverted group, the cumulative incidence of HCC were 7 (2.2%), 18 (5.6%), and 27 (8.4%) at 2, 5, and 8 years, respectively (*p* = 0.529). 

#### 3.5.5. HCC Development According to Antiviral Agents

Of the 610 patients, 400, 150, and 60 patients were treated with ETV, TDF, or a combination of both. 

ALT normalization was noted in 271 (67.8%) of 400, 89 (59.3%) of 150, and 37 (61.7%) of 60 patients treated with ETV, TDF, or a combination of both (*p* = 0.156). When we compared the incidence of HCC in patients treated with ETV or TDF, there was no significant difference in the risk of HCC when using TDF compared to ETV (aHR 0.531, 95% CI 0.258–1.356, *p* = 0.215). In addition, when we compared the HCC incidence in patients treated with a combination of both drugs with ETV or TDF mono-treated patients, HCC incidence was lower either in ETV mono-treated (aHR 0.441, 95% CI 0.204–0.954, *p* = 0.038) or TDF mono-treated patients (aHR 0.261, 95% CI 0.093–0.733, *p* = 0.011) (Table 3). It was found that each group that used a single drug had a lower risk of HCC occurrence than those using both drugs. 

#### 3.5.6. HCC Development According to Naïve and Non-Naïve Patients

Of the 610 patients, 521 (85.4%) were naïve to antiviral treatment, and 89 (14.6%) had previously received antiviral treatment (adefovir (*n* = 2), clevudine (12), pegylated interferon α (15), telbivudine (4), and lamivudine (56)).

ALT normalization occurred in 337 (64.7%) of 521 patients in the naïve group and 60 (67.4%) of 89 patients in the non-naïve group. There was no significant difference in ALT normalization between naïve and non-naïve groups (*p* = 0.617). 

A total of 56 (10.7%) of 521 naïve patients developed HCC. The cumulative incidence of HCC in these patients were 16 (3.1%), 44 (8.4%), and 54 (10.4%) at 2, 5, and 8 years, respectively. Nine (10.1%) out of 89 non-naïve patients developed HCC. In non-naïve patients, the cumulative incidence of HCC were 2 (2.2%), 4 (4.5%), and 9 (10.1%) at 2, 5, and 8 years, respectively (*p =* 0.567). 

### 3.6. Risk Factors for ALT Non-Normalization by Logistic Regression Analysis

In univariate analysis, age, baseline platelet, AST, ALT, DNA level, cholesterol, BMI, alcohol use, and presence of liver cirrhosis were related to ALT normalization (Table 4). In multivariate analysis, BMI (adjusted odds ratio (aOR) 1.162, 95% CI 1.095–1.232, *p* < 0.001), cholesterol (aOR 1.006, 95% CI 1.001–1.012, *p* = 0.028), presence of liver cirrhosis (aOR 2.551, 95% CI 1.632–3.990, *p* < 0.001) at baseline were significantly associated with ALT normalization.

### 3.7. Risk Factors for HCC by Cox Regression Analysis

In the univariate analysis, male sex, age, baseline platelet, AST, ALT, albumin, prothrombin time (PT), cholesterol, HBV DNA level, HBeAg, alcohol use, diabetes mellitus (DM), hypertension (HTN), presence of liver cirrhosis, and ALT normalization at 1 year of treatment were associated with HCC development (Table 5).

In multivariate analysis, male sex (aHR 2.782, 95% CI 1.399–5.529, *p =* 0.004), age (aHR 1.080, 95% CI 1.048–1.113, *p* < 0.001), baseline platelet (aHR 0.992, 95% CI 0.985–0.998, *p =* 0.011), alcohol use (aHR 2.105, 95% CI 1.194–3.711, *p =* 0.010), and presence of liver cirrhosis at baseline (aHR 2.955, 95% CI 1.591–5.486, *p =* 0.001) were significantly associated with HCC risk. ALT non-normalization after 1 year of treatment (aHR 2.641, 95% CI 1.524–4.577, *p* = 0.001) was independently associated with HCC risk in a multivariate time-dependent Cox regression analysis. 

## 4. Discussion

Worldwide, the prevalence of HBV has been decreasing since the introduction of neonatal HBV vaccination [4], but still many patients with chronic HBV have the potential to develop cirrhosis, liver-related diseases, and HCC. With the use of potent antiviral agents, virologic, serologic, and biochemical responses can be expected in patients with chronic HBV; these are major surrogate parameters to estimate the antiviral response [16,17].

Among the parameters, virologic and serologic responses are major indicators of antiviral response. In patients with non-cirrhotic CHB treated with oral antivirals, cessation of the drug can be expected if HBV DNA is suppressed to undetectable levels and HBeAg seroconversion occurs [9]. However, biochemical response, defined as ALT normalization, is relatively less important than other indicators. Consequently, biochemical responses usually do not change the treatment course. However, recently, clinical results have reported that ALT normalization may have an important association with the risk factors for HCC development [12,18]. The aim of our study was to determine the correlation of ALT normalization within 12 months through the administration of potent antiviral drugs that may affect survival, liver-related events, and HCC development.

As shown in our results, patients with or without ALT normalization after a year of potent antivirals were well balanced after propensity score matching. When we compared the incidence of liver cirrhosis and liver-related events, and mortality, they did not differ between patients with and without ALT normalization. This may be because of the relatively short period of follow-up or a small number of patients. In contrast with the results of our study, the occurrence of cirrhosis according to ALT normalization was not reported in other studies. In a study by Wong et al., composite hepatic events, including HCC were identified as primary end points, and significant differences were observed according to ALT normalization. In their study, ALT normalization at 12 months was significantly associated with fewer hepatic events although the number of hepatic events excluding HCC was small [12]. In another study from Korea, mortality or liver transplantation rate according to ALT normalization was significantly lower in the ALT normalization group [18]. In our study, mortality rate was low in patients group with ALT normalization compared to that of those with non-normalization, but it was not statistically significant.

When we compared the development of HCC according to ALT normalization, there was a significant difference in the incidence of HCC between the ALT normalization group and the non-ALT normalization group. Among the 610 patients, the group with ALT normalization (Gr.1) had a significantly lower cumulative incidence of HCC than the ALT non-normalization group (Gr.2). Even after propensity matching, Gr.1 had a significantly lower cumulative incidence of HCC than Gr.2. A large study conducted in Hong Kong also supported these findings [12]. A total of 21,182 patients with CHB were enrolled, and composite hepatic events including HCC were the primary endpoints. At 3, 6, 9, and 12 months of treatment, the incidence of cumulative composite hepatic events was significantly lower in the ALT normalization group than in the non-normalization group [12]. In addition, a study involving 4639 patients in Korea also found that ALT normalization at the first and second years of treatment was significantly associated with lower HCC risk [18].

Cirrhosis is a well-established risk factor for HCC, but the risk of HCC is not constant because the etiologies of cirrhosis vary [19]. When we divided the patients according to the presence or absence of cirrhosis at baseline, a significant difference in the incidence of HCC was observed only in the group without cirrhosis. Although it cannot be explained accurately, patients without cirrhosis had higher HBV DNA levels in the first year of treatment and higher baseline ALT and cholesterol levels than those with cirrhosis, which may support our findings. Factors such as the BCP T1762/A1764 mutation, high viral load [20], and co-existence of nonalcoholic fatty liver disease (NAFLD) [21] are known to contribute to the increased risk of HCC in patients with non-cirrhotic chronic hepatitis B. In a study by West J et al., they examined the cumulative incidence of HCC in a representative population of people with cirrhosis of the liver of varying etiologies [22]. The 10-year predicted cumulative incidence estimates of HCC varied depending on the etiology of cirrhosis. Estimates of each etiology were 1.2% for alcohol, 3.2% for autoimmune or metabolic disease, and 1.1% for cryptogenic, but the highest was 4.0% for chronic viral hepatitis. Thus, the HCC cause by CHB-induced liver cirrhosis can be considered to exceed the effect of normalization or non-normalization levels of ALT.

In our study, no significant difference in HCC development according to the presence or absence of HBeAg seroconversion was observed between the two groups. HBeAg seroconversion is one of the important clinical endpoints of antiviral treatment in patients with CHB and has been known as a beneficial clinical outcome [23]. Some studies have shown that patients undergoing HBeAg seroconversion have a lower risk of HCC than those that are consistently positive for HBeAg [17]. However, a meta-analysis study described that HCC can still occur after HBeAg seroconversion [24]. Covalently closed circular DNA (cccDNA) in the nucleus of HBV-infected cells cannot be eliminated by present treatments and may result in persistence and relapse [25]. The integrated DNA can express HBV antigens and promotes immune-mediated responses. The seroconversion of HBeAg is one of the results of this immune-mediated response, but immune mechanism involves immune cells and factors such as HBV-specific CD8+ T cells, HBV-non-specific CD8+, CD4+ T cells and B cells, NK/NKT cells, Kupffer cells, and hepatic stellate cells [26]. These cause cumulative immune mediated hepatocyte damage in the HBV-infected liver and lead to HCC development. Thus, as a surrogate marker for antiviral therapy of CHB, HBeAg seroconversion may not be particularly suitable for HCC surveillance.

Since the introduction of potent antiviral agents against HBV, there have been many controversial reports on the incidence of HCC between ETV and TDF. In a cohort study of 24,156 Korean patients with CHB, TDF treatment had a significantly lower risk of HCC than ETV treatment [27]. However, another multicenter study from South Korea concluded that HCC risk was not statistically different between patients treated with ETV and those treated with TDF [28]. In our study, the risk of HCC was not statistically different between ETV and TDF. Because TDF has been available in Korea since 2012, most patients were ETV users in the initial data collection period. For this reason, the ETV group had more patients than the TDF group in the entire pool. Regarding tenofovir, in a study comparing TDF and tenofovir alafenamide fumarate (TAF), patients treated with TAF achieved ALT normalization more rapidly during treatment than those treated with TDF despite similar viral suppression [29,30]. In a randomized, double-blind, phase three, non-inferiority trial comparing TAF and TDF administration in HBeAg-positive or HBeAg-negative patients with CHB, there was a significant difference in ALT normalization at 48 weeks between the two drugs based on the AASLD criteria [29,30]. At present, although strong evidence to support the preference of one drug to another is lacking, the clinical impact of early ALT normalization on the occurrence of HCC should be considered when choosing antivirals in patients with CHB. In addition to metabolic syndrome, long-term comparative studies on antivirals, such as TAF and TDF, are expected to determine whether these drugs will contribute to the occurrence of HCC. 

In our study, the adjusted HR of HCC was significantly higher in patients treated with a combination of ETV and TDF than in those treated with a single drug. In the group using both drugs, the HBV DNA non-detection rate at 1 year of treatment was significantly lower than that in the group with a single drug, and more patients experienced previous CHB treatment. Most patients who used both drugs underwent a switch in their drug because they did not get a biochemical or viral response to the drug previously used. It was thought that a delayed biochemical or viral response would affect the development of HCC [31]. 

Most other studies analyzed only naïve patients with antiviral drugs, but in this study, both groups were analyzed. There was no significant difference in ALT normalization or HCC incidence according to the treatment experience group or naïve group.

Our study also showed that high BMI, high cholesterol, and presence of cirrhosis at baseline are associated with ALT non-normalization. Metabolic syndrome is a risk factor for NAFLD [32]. Also, metabolic syndrome is an independent risk factor for liver cirrhosis in CHB [33]. If the elevation of ALT persists despite treatment in patients with chronic HBV, the coexistence of NAFLD and metabolic syndrome should be considered [34]. 

After multivariate analysis, we identified that ALT normalization within a year of potent antiviral treatment is a strong indicator of HCC development. In addition to previously known risk factors for HCC incidence, including male sex (aHR 2.78), old age (aHR 1.08), cirrhosis (aHR 2.95), and alcohol (aHR 2.10), ALT non-normalization within 1 year had a strong aHR of 2.64 for HCC development. In addition, ALT normalization was an important factor in the occurrence of HCC even when propensity matching was performed to correct factors that may affect ALT normalization, such as metabolic syndrome.

The retrospective design of this study has limitations. This was a single-center study, and the number of patients was relatively limited. The patients consisted only of Koreans. However, this study has several clinical implications. Factors that influence ALT normalization include cholesterol, BMI, and presence of cirrhosis at baseline, were corrected and analyzed through propensity score matching. Regarding HCC occurrence, we analyzed several factors, including HBeAg seroconversion, presence or absence of cirrhosis, naïve or experienced patients, and type of drugs. Other studies have mainly analyzed naïve patients, but we also analyzed the treatment experience group, which may have clinical implication. However, since most of the non-naïve patients received previous medications at other hospitals, it was not possible to accurately determine the duration of previous treatment for CHB. This is a limitation because information about the entire treatment period, which can affect the treatment response, was not available in the study.

In conclusion, early ALT normalization after administration of potent antiviral drugs was related to the occurrence of HCC. When ALT normalization is delayed during treatment with CHB patients, concomitant factors such as fatty liver should be identified, and efforts such as correcting complications and metabolic diseases or modifying antiviral treatment strategies are required. If ALT normalization is delayed despite these efforts, HCC surveillance should be carefully conducted. 

## Figures and Tables

**Table 1:** Comparison of demographic characteristics of patients with CHB -with ALT normalization (Gr.1) or –without ALT normalization (Gr.2) within 1 year of potent antiviral treatment.

Characteristics	Total Patients (*n* = 610)	Gr.1 (*n* = 397)	Gr.2 (*n* = 213)	*p*-Value
Male gender (*n*, %)	382 (62.6)	252 (63.5)	130 (61.0)	0.552
Age (years)	47 (IQR 39–55)	47 (IQR 38–54)	49 (IQR 43–55)	0.008 *
Creatinine (mg/dL)	1.00 (IQR 0.89–1.00)	1.00 (IQR 0.83–1.00)	1.00 (IQR 1.00–1.00)	0.317
Platelet (×10^3^/uL)	148 (IQR 109–187)	153 (IQR 117.5–190)	142 (IQR 102–176)	0.007 *
Albumin (g/dL)	4.0 (IQR 4.0–4.0)	4.0 (IQR 4.0–4.0)	4.0 (IQR 4.0–4.0)	0.343
Total bilirubin (mg/dL)	1.00 (IQR 1.00–1.21)	1.00 (IQR 1.00–1.29)	1.00 (IQR 1.00–1.00)	0.224
ALT (IU/L)	113 (IQR 66–227)	141 (IQR 82.0–323.5)	81 (IQR 53.5–134.5)	<0.001 *
AST (IU/L)	87 (IQR 56–169)	108 (IQR 65.00–216.00)	66 (IQR 49–105)	<0.001 *
Positive HBeAg (*n*, %)	379 (62.1)	244 (61.5)	135 (63.4)	0.641
HBV DNA (IU/mL)	5.64 × 10^6^ (IQR 0.55 × 10^6^–91.33 × 10^6^	7.37 × 10^6^ (IQR 0.76 × 10^6^–127.68 × 10^6^	2.44 × 10^6^ (IQR 0.23 × 10^6^–39.43 × 10^6^	<0.001 *
Naïve/non-Naïve Antiviral therapy (*n*, %)	521 (85.4)/89 (14.6)	337 (84.9)/60(15.1)	184 (86.4)/29(13.6)	0.617
Presence/Absence cirrhosis (*n*, %)	186 (30.5)/424 (69.5)	89 (22.4)/308 (77.6)	97 (45.5)/116 (54.5)	<0.001 *
ETV/TDF/Both (*n*, %)	400(65.5)/156 (25.6)/54(8.9)	271(68.3)/89(22.4)/37(9.3)	129(60.6)/61(28.6)/23(10.8)	0.154
Alcohol use (*n*, %)	149 (24.4)	88 (22.2)	61 (28.6)	0.076
HTN (*n*, %)	95 (15.6)	55 (13.9)	40 (18.8)	0.110
DM (*n*, %)	67 (11.0)	39 (9.8)	28 (13.1)	0.211
BMI	24.0 (IQR 22–26)	23.2 (IQR 21.1–26.0)	25.0 (IQR 23.0–27.0)	<0.001 *
Follow-up duration (month)	86 (IQR 35–94)	87 (IQR 35–95)	83 (IQR 32.5–93.5)	0.178

ALT, alanine aminotransferase; AST, aspartate aminotransferase; HBeAg, hepatitis B e antigen; HBV DNA, hepatitis B virus deoxyribonucleic acid; ETV, entecavir; TDF, tenofovir disoproxil fumarate; DM, diabetes mellitus; HTN, hypertension; BMI, body mass index. * Denote the significant values.

**Table 2:** Baseline characteristics of patients with CHB-with ALT normalization (Gr.1) or –without ALT normalization (Gr.2) within 1 year of potent antiviral treatment after propensity matching.

Characteristics	Propensity Matching		
	Gr. 1 (*n* = 189)	Gr. 2 (*n* = 189 )	*p*-Value
Male gender (*n*, %)	124 (65.6%)	117 (61.9%)	0.454
Age (years)	48 (IQR 41–56)	49 (IQR 42–55)	0.760
Creatinine (mg/dL)	1.00 (IQR 1.00–1.27)	1.00 (IQR 0.97–1.00)	0.939
Platelet (×10^3^/uL)	143 (IQR 97–183)	142 (IQR 103–176)	0.885
Albumin (g/dL)	4.00(IQR 3.90–4.00)	4.00 (IQR 4.00–4.00)	0.311
Total bilirubin (mg/dL)	1.00 (IQR 1.00–1.27)	1.00 (IQR 1.00–1.00)	0.726
ALT (IU/L)	100 (IQR 59.5–192.0)	82 (IQR 54–136)	0.315
AST (IU/L)	89 (IQR 58–150)	65 (IQR 48–105.5)	0.171
Positive HBeAg (*n*, %)	110 (58.2%)	121 (64.0%)	0.246
HBV DNA (IU/mL)	5.04 × 10^6^ (IQR 0.51 × 10^6^–110.77 × 10^6^)	3.56 × 10^6^ (IQR 0.23 × 10^6^–41.12 × 10^6^)	0.096
Naïve/non-Naïve Antiviral therapy (*n*, %)	165 (87.3%)/24 (12.7%)	161 (85.2%)/28 (14.8%)	0.550
Presence/Absence cirrhosis (*n*, %)	78 (41.3)/111(58.7)	82 (43.4)/107(56.6)	0.677
ETV (*n*, %)	135 (71.4)	113 (59.8)	0.017 *
TDF (*n*, %)	38 (20.1)	57 (30.1)	0.024
Both (*n*, %)	16 (8.5)	19 (10.1)	0.594
Alcohol use (*n*, %)	48 (25.4%)	49 (25.9%)	0.906
HTN (*n*, %)	35 (18.5%)	33 (17.5%)	0.789
DM (*n*, %)	26 (13.8%)	23 (12.2%)	0.646
BMI	24.9 (IQR 23–27)	24 (IQR 23–27)	0.787
Follow-up duration (month)	90 (IQR 41.5–97.0)	83 (IQR 32.5–93.5)	0.017 *

ALT, alanine aminotransferase; AST, aspartate aminotransferase; HBeAg, hepatitis B envelope antigen; HBV DNA, hepatitis B virus deoxyribonucleic acid; ETV, entecavir; TDF, tenofovir disoproxil fumarate; DM, diabetes mellitus; HTN, hypertension; BMI, body mass index. * Denote the significant values.

**Table 3:** HCC risk in a multivariate time-dependent Cox regression analysis according to antiviral agents use.

Antiviral Agents.	No HCC (*n* = 545)	HCC (+) (*n* = 65)	aHR (95% CI)	*p*-Value
**ETV vs. TDF**				
ETV only (*n* = 400)	353/400 (88.2%)	47/400 (11.8%)	1	-
TDF only (*n* = 150)	149/150 (95.5%)	7/150 (4.5%)	0.531 (0.258–1.356)	0.215
**Both use vs. ETV only**				
Both (*n* = 60)	43/60 (79.6%)	11/60 (20.4%)	1	-
ETV only (*n* = 400)	353/400 (88.2%)	47/400 (11.8%)	0.441 (0.204–0.954)	0.038 *
**Both use vs. TDF only**				
Both (*n* = 60)	43/60 (79.6%)	11/60 (20.4%)	1	-
TDF only (*n* = 150)	149/150 (95.5%)	7/150 (4.5%)	0.261 (0.093–0.733)	0.011 *

HCC, hepatocellular carcinoma; aHR, adjusted hazard ratio; ETV, entecavir; TDF, tenofovir disoproxil fumarate. Sex, age, baseline platelet, AST, ALT, albumin, PT, cholesterol, HBV DNA titer, HBeAg, anti-HBe Ab, alcohol, DM, HTN, and ALT normalization one year after adjustment in the Cox regression model. * Denote the significant values.

**Table 4:** Univariate and multivariate analyses for ALT non-normalization.

Baseline Variables	Univariate OR (95% CI)	*p*-Value	Multivariate aOR (95% CI)	*p*-Value
Male sex	0.901 (0.640–1.270)	0.552		
Age	1.018 (1.003–1.033)	0.019 *	1.005 (0.987–1.023)	0.609
Hb	1.025 (0.932–1.127)	0.611		
PLT	0.997 (0.994–1.000)	0.060 *	1.000 (0.996–1.004)	0.973
AST	0.998 (0.996–0.999)	0.001 *	0.999 (0.997–1.001)	0.476
ALT	0.998 (0.997–0.999)	0.001 *	0.999 (0.997–1.001)	0.328
Total bilirubin	0.933 (0.848–1.027)	0.156		
Albumin	0.844 (0.584–1.220)	0.368		
BUN	0.994 (0.958–1.031)	0.743		
Creatinine	0.965 (0.638–1.458)	0.864		
PT (INR)	1.764 (0.789–3.946)	0.167		
Cholesterol	1.006 (1.001–1.011)	0.020 *	1.006 (1.001–1.012)	0.028 *
HBV DNA	0.767 (0.675–0.872)	0.001 *	0.868 (0.751–1.002)	0.053
HBeAg	1.085 (0.769–1.531)	0.641		
Anti-HBeAb	0.982 (0.702–1.373)	0.915		
AFP	0.999 (0.997–1.001)	0.337		
BMI	1.176 (1.113–1.243)	0.001 *	1.162 (1.095–1.232)	<0.001 *
Alcohol use	1.409 (0.964–2.060)	0.077 *	1.312(0.864–1.993)	0.202
DM	1.389 (0.829–2.330)	0.212		
HTN	1.438 (0.920–2.247)	0.111		
Presence cirrhosis	2.894 (2.022–4.141)	0.001*	2.551 (1.632–3.990)	<0.001 *
Non-naïve antiviral therapy	0.885 (0.549–1.428)	0.617		

OR, odds ratio; aOR, adjusted odds ratio; Hb, hemoglobin; PLT, platelet; AST, aspartate transaminase; ALT, alanine transaminase; BUN, blood urea nitrogen; PT, prothrombin time; INR, international normalized ratio; HBV DNA, hepatitis B virus deoxyribonucleic acid; HBeAg, hepatitis B envelope antigen; anti-HBe Ab, anti-hepatitis B envelope antibody; AFP, α-fetoprotein; BMI, body mass index; DM, diabetes mellitus; HTN, hypertension. * Denote the significant values.

**Table 5:** Univariate and multivariate analyses for HCC by Cox regression analysis.

Baseline Variables	Univariate HR (95% CI)	*p* Value	Multivariate aHR (95% CI)	*p*-Value
Male sex	1.892 (1.076–3.327)	0.027 *	2.782 (1.399–5.529)	0.004 *
Age	1.079 (1.055–1.104)	<0.001 *	1.080 (1.048–1.113)	<0.001 *
Hb	0.962 (0.841–1.101)	0.576		
PLT	0.987 (0.982–0.992)	<0.001 *	0.992 (0.985–0.998)	0.011 *
AST	0.997 (0.995–1.000)	0.040 *	0.999 (0.996–1.002)	0.502
ALT	0.997 (0.995–0.999)	0.005 *	1.000 (0.999–1.002)	0.901
Total bilirubin	0.957 (0.825–1.111)	0.567		
Albumin	0.502 (0.317–0.796)	0.003 *	0.647 (0.334–1.254)	0.198
BUN	1.018 (0.977–1.062)	0.394		
Creatinine	1.004 (0.597–1.689)	0.987		
PT (INR)	2.611 (1.012–6.736)	0.047 *	0.830 (0.217–3.171)	0.786
Cholesterol	0.992 (0.985–1.000)	0.044 *	0.998 (0.990–1.007)	0.728
HBV DNA	0.759 (0.640–0.900)	0.002 *	0.986 (0.783–1.241)	0.905
HBeAg	0.608 (0.374–0.989)	0.045 *	1.033 (0.442–2.416)	0.940
Anti-HBeAb	1.706 (1.046–2.782)	0.032 *	1.799 (0.755–4.289)	0.185
AFP	0.999 (0.997–1.002)	0.692		
BMI	1.034 (0.962–1.112)	0.366		
Alcohol use	2.534 (1.550–4.141)	<0.001 *	2.105 (1.194–3.711)	0.010 *
DM	1.846 (1.005–3.390)	0.048 *	0.757 (0.400–1.436)	0.394
HTN	2.460 (1.453–4.167)	0.001 *	1.448 (0.801–2.620)	0.221
Presence of cirrhosis	6.797 (3.909–11.819)	<0.001 *	2.955 (1.591–5.486)	0.001 *
Non-naïve antiviral therapy	0.815 (0.403–1.648)	0.568		
**On-treatment variables**				
ALT non-normalization	2.753 (1.684–4.499)	<0.001 *	2.641 (1.524–4.577)	0.001 *
DNA titer < 20 after 1 year	1.126 (0.677–1.873)	0.649	1.355 (0.730–2.515)	0.335

HCC, hepatocellular carcinoma; aHR, adjusted hazard ratio; Hb, hemoglobin; PLT, platelet; AST, aspartate transaminase; ALT, alanine transaminase; BUN, blood urea nitrogen; PT, prothrombin time; INR, international normalized ratio; HBV DNA, hepatitis B virus deoxyribonucleic acid; HBeAg, hepatitis B envelope antigen; anti-HBe Ab, anti-hepatitis B envelope antibody; AFP, alpha-fetoprotein; BMI, body mass index; DM, diabetes mellitus; HTN, hypertension. * Denote the significant values.

## Data Availability

The data presented in this study are available on request from the corresponding author.
